# Supplementary material for: Dental caries and associated factors among patients visiting Shashamane Comprehensive Specialized Hospital
Source: PLoS One. 2022 Mar 3;17(3):e0265000. doi: 10.1371/journal.pone.0265000 (PMC8893641; doi:10.1371/journal.pone.0265000)
Supplement: Attachment — Submitted filename: Response to reviewers.docx [file pone.0265000.s001.docx]

**Response to comments**

Journal Requirements:

When submitting your revision, we need you to address these additional requirements.

1. Please ensure that your manuscript meets PLOS ONE's style requirements, including those for file naming. The PLOS ONE style templates can be found at
   https://journals.plos.org/plosone/s/file?id=wjVg/PLOSOne_formatting_sample_main_body.pdf and 
   <https://journals.plos.org/plosone/s/file?id=ba62/PLOSOne_formatting_sample_title_authors_affiliations.pdf>

Response: Thank you for the comment; we have checked and corrected according it to the guideline.

2. You indicated that you had ethical approval for your study. In your Methods section, please ensure you have also stated whether you obtained consent from parents or guardians of the minors included in the study or whether the research ethics committee or IRB specifically waived the need for their consent.

Response: We have moved Ethical consideration to Method section. We have also included a statement which indicates about parent or guardian consent for children.

3. Please include additional information regarding the survey or questionnaire used in the study and ensure that you have provided sufficient details that others could replicate the analyses. For instance, if you developed a questionnaire as part of this study and it is not under a copyright more restrictive than CC-BY, please include a copy, in both the original language and English, as Supporting Information

Response: The questionnaire (data collection tool) was adapted with modification from the WHO survey tool as indicated in section ‘data collection’ . We have included the reference for it.

4. Thank you for stating the following financial disclosure:

not applicable

At this time, please address the following queries:

a) Please clarify the sources of funding (financial or material support) for your study. List the grants or organizations that supported your study, including funding received from your institution. 
b) State what role the funders took in the study. If the funders had no role in your study, please state: “The funders had no role in study design, data collection and analysis, decision to publish, or preparation of the manuscript.”
c) If any authors received a salary from any of your funders, please state which authors and which funders.
d) If you did not receive any funding for this study, please state: “The authors received no specific funding for this work.”

Please include your amended statements within your cover letter; we will change the online submission form on your behalf.

5. Thank you for stating the following in your Competing Interests section:

NO authors have competing interests
 
Please complete your Competing Interests on the online submission form to state any Competing Interests. If you have no competing interests, please state "The authors have declared that no competing interests exist.", as detailed online in our guide for authors at http://journals.plos.org/plosone/s/submit-now 
 This information should be included in your cover letter; we will change the online submission form on your behalf.

6. Your ethics statement should only appear in the Methods section of your manuscript. If your ethics statement is written in any section besides the Methods, please move it to the Methods section and delete it from any other section. Please ensure that your ethics statement is included in your manuscript, as the ethics statement entered into the online submission form will not be published alongside your manuscript.

Response: We moved the ethical consideration to the method section as mentioned above.

[Note: HTML markup is below. Please do not edit.]

Reviewers' comments:

Reviewer's Responses to Questions

**Comments to the Author**

1. Is the manuscript technically sound, and do the data support the conclusions?

The manuscript must describe a technically sound piece of scientific research with data that supports the conclusions. Experiments must have been conducted rigorously, with appropriate controls, replication, and sample sizes. The conclusions must be drawn appropriately based on the data presented.

Reviewer #1: Yes

Reviewer #2: Partly

2. Has the statistical analysis been performed appropriately and rigorously?

Reviewer #1: I Don't Know

Reviewer #2: N/A

3. Have the authors made all data underlying the findings in their manuscript fully available?

The [PLOS Data policy](http://www.plosone.org/static/policies.action#sharing) requires authors to make all data underlying the findings described in their manuscript fully available without restriction, with rare exception (please refer to the Data Availability Statement in the manuscript PDF file). The data should be provided as part of the manuscript or its supporting information, or deposited to a public repository. For example, in addition to summary statistics, the data points behind means, medians and variance measures should be available. If there are restrictions on publicly sharing data—e.g. participant privacy or use of data from a third party—those must be specified.

Reviewer #1: No

Reviewer #2: Yes

Response: We included all the data collected in the result section in the form of tables and text.

4. Is the manuscript presented in an intelligible fashion and written in standard English?

PLOS ONE does not copyedit accepted manuscripts, so the language in submitted articles must be clear, correct, and unambiguous. Any typographical or grammatical errors should be corrected at revision, so please note any specific errors here.

Reviewer #1: Yes

Reviewer #2: No

*Response:* We have revised the English as shown in the manuscript with track change.

5. Review Comments to the Author

Please use the space provided to explain your answers to the questions above. You may also include additional comments for the author, including concerns about dual publication, research ethics, or publication ethics. (Please upload your review as an attachment if it exceeds 20,000 characters)

Reviewer #1: Abstract : within 300 words

Response: We would like to thank you for thoroughly reviewing the manuscript and giving us valuable comments. The number of words in the abstract is less than 300.

Line no 16-17: In Ethiopia, prevention and treatment of oral health-related illness had been given little attention line no 33: This study revealed a high prevalence of dental carries

Response: The comment is not clear. The statement mentioned in line number 16-17 is for introduction purposes whereas the one mentioned in line number 33 is our finding.

Line no 36-37: Key words should be arranged alphabetically

Response: According to the comment, Keywords are arranged alphabetically as shown in the track change.

Line no 52 and 53: Even though there is improvement in maintaining oral health worldwide, the problem is still predominant in low-income countries. Also reference for this line.
Response: Based on the comment we have added the reference.

Line no 204-6 : rephrasing required.

Response: Thank you for the comment; we rephrased it as shown in the track change.

Recommended read:
1. Zewdu T, Abu D, Agajie M, Sahilu T. Dental caries and associated factors in Ethiopia: systematic review and meta-analysis. Environ Health Prev Med. 2021 Feb 12;26(1):21. doi: 10.1186/s12199-021-00943-3. PMID: 33579186; PMCID: PMC7881546.
2. Teshome A, Andualem G, Derese K. Dental Caries and Associated Factors Among Patients Attending the University of Gondar Comprehensive Hospital Dental Clinic, North West Ethiopia: A Hospital-Based Cross-Sectional Study. Clin Cosmet Investig Dent. 2020;12:191-198
https://doi.org/10.2147/CCIDE.S247179
3. Anguach Shitie, Rahel Addis, Abebe Tilahun, Wassie Negash, "Prevalence of Dental Caries and Its Associated Factors among Primary School Children in Ethiopia", International Journal of Dentistry, vol. 2021, Article ID 6637196, 7 pages, 2021. <https://doi.org/10.1155/2021/6637196>

Response: Thank you for literatures. We have considered them in the discussion section.

Reviewer #2: Thank you for the manuscript.

First of all, the anonymity of survey data is not mentioned. Also, what steps did the authors took to safeguard the patient identity.

Response: Thank you for the comment, based on the comment we have included the following in ethical consideration section ‘Written informed consent was also obtained from parents or guardians for children participants. Any information which indicates the Identity of the participants was removed from data collection tool. All information collected from participants was kept confidential’

In 104 line it was mentioned that a structured questionnaire was modified from WHO oral health survey. Could you please elaborate on that? Furthermore, the exact questionnaire should be presented in the manuscript.

Response: For the current study we have adapted a survey tool from WHO (we cited it), We have used the adapted questionnaire to collect socio-demographic characteristics, dietary habits, and factors that affect dental health as shown in the track change. It may not be appropriate for the whole survey tool as it is not ours.

How many evaluators were there? Were they calibrated?

Response: This comment is not clear. To confirm dental caries we followed the procedure recommended by WHO as indicated in the manuscript.

The uniqueness of the population studied with respect to the other African studies was not discussed in detail. The introduction does not describe in detail the main issue of the paper.

Response: In the discussion, we have mentioned that the ‘study population’ is one reason for the difference in the finding as shown in the track change.

lines 234-239 - there is no literature reference

Response: The reason we did not include the reference is because it is our comments

line 116 - if the tooth was extracted not at the same clinic and the authors did not have any records of that, how did authors determine the reason of tooth extraction?

Response: We identified by asking past history of participants.


The authors said that the response rate was 100% and it is hard to believe that all of 288 patients have fully filled the questionnaires.

Response: All participants approached agreed to take part in the study.

lines 166 - 167 - the authors should change the words into numbers, there has to be consistency throughout the manuscript.

Response: We have corrected accordingly-changed words into numbers


There cannot be a p value equal to 0.000.

Response: It is the value that SPSS gave us during analysis; we have consulted with the statistician and corrected it as <0.001.


What do the authors consider as a statistically significant result? When COR is <0.05, AOR <0.05 or both?

Response: As indicated in data analysis section, a P-value <0.05 and AOR were considered to determine significant association.

The discussion section is descriptive, and does not really discuss the results. The results were compared to other studies, however, there were no suggestions how to solve the problems. In the conclusions there are new ideas like integrating promotion of oral health services that has been never mentioned in the discussion, therefore, the conclusions does not really conclude the manuscript. Furthermore, when the results are compared to other studies - where the studies conducted in other countries in the same design? A specification is needed.

Response: Based on the comment we have added the following at the end of the discussion ‘The burden of dental caries can be reduced by providing proper health education on how to keep oral hygiene and regular visit of dental clinic’. We have also included the nature of study (in the discussion section) we have used for comparison as indicated in the manuscript with track change.

-We have removed the content claimed form the conclusion section.

The quality of the language could be better. The authors change past and present times a few times throughout the manuscript.

Response: We have revised and corrected all of them as indicated in the manuscript with track change.

line 43 - develops instead of develop (corrected)
56 - ‘to’ is missing?
69 - keeping in mind - lowercase letter should be changed in to uppercase at the beginning of the sentence (corrected)
179- index is not a DMF, but DMFT, I would recommend using the newest D3MFT index (corrected)
183- didn’t into did not (corrected)
211- MDFT index (corrected)
Throughout the manuscript different forms of words used - ‘physical examination’ vs ‘intraoral examination’ and they should be unified.

Response: We have corrected all of them.

6. PLOS authors have the option to publish the peer review history of their article ([what does this mean?](https://journals.plos.org/plosone/s/editorial-and-peer-review-process#loc-peer-review-history)). If published, this will include your full peer review and any attached files.


If you choose “no”, your identity will remain anonymous but your review may still be made public.


**Do you want your identity to be public for this peer review?** For information about this choice, including consent withdrawal, please see our [Privacy Policy](https://www.plos.org/privacy-policy).

Reviewer #1: No

Reviewer #2: No

[NOTE: If reviewer comments were submitted as an attachment file, they will be attached to this email and accessible via the submission site. Please log into your account, locate the manuscript record, and check for the action link "View Attachments". If this link does not appear, there are no attachment files.]

While revising your submission, please upload your figure files to the Preflight Analysis and Conversion Engine (PACE) digital diagnostic tool, <https://pacev2.apexcovantage.com/>. PACE helps ensure that figures meet PLOS requirements. To use PACE, you must first register as a user. Registration is free. Then, login and navigate to the UPLOAD tab, where you will find detailed instructions on how to use the tool. If you encounter any issues or have any questions when using PACE, please email PLOS at [figures@plos.org](mailto:figures@plos.org). Please note that Supporting Information files do not need this step.
