# Supplementary material for: Dental caries and associated factors among patients visiting Shashamane Comprehensive Specialized Hospital
Source: PLoS One. 2022 Mar 3;17(3):e0265000. doi: 10.1371/journal.pone.0265000 (PMC8893641; doi:10.1371/journal.pone.0265000)
Supplement: Attachment — Submitted filename: Response to reviewers.docx [file pone.0265000.s002.docx]

Additional Editor Comments:

Please take into account the comments of reviewer 2 when writing the discussion section.

Response: Thank you for the comment, we have included study design for studies we have used for comparison we have omitted “Khat is commonly used in Ethiopia for social and pleasure purposes. Also, certain occupational groups like long-distance truck drivers and students during examination time have a high tendency to consume Khat.” From the discussion section. In the previous comment we have been asked to provide reference; however, since it is our idea we are unable to provide reference as a result we omitted it.


[Note: HTML markup is below. Please do not edit.]

Reviewers' comments:

Reviewer's Responses to Questions

**Comments to the Author**

1. If the authors have adequately addressed your comments raised in a previous round of review and you feel that this manuscript is now acceptable for publication, you may indicate that here to bypass the “Comments to the Author” section, enter your conflict of interest statement in the “Confidential to Editor” section, and submit your "Accept" recommendation.

Reviewer #1: All comments have been addressed

Reviewer #2: All comments have been addressed

2. Is the manuscript technically sound, and do the data support the conclusions?

The manuscript must describe a technically sound piece of scientific research with data that supports the conclusions. Experiments must have been conducted rigorously, with appropriate controls, replication, and sample sizes. The conclusions must be drawn appropriately based on the data presented.

Reviewer #1: Yes

Reviewer #2: Partly

3. Has the statistical analysis been performed appropriately and rigorously?

Reviewer #1: Yes

Reviewer #2: I Don't Know

4. Have the authors made all data underlying the findings in their manuscript fully available?

The [PLOS Data policy](http://www.plosone.org/static/policies.action#sharing) requires authors to make all data underlying the findings described in their manuscript fully available without restriction, with rare exception (please refer to the Data Availability Statement in the manuscript PDF file). The data should be provided as part of the manuscript or its supporting information, or deposited to a public repository. For example, in addition to summary statistics, the data points behind means, medians and variance measures should be available. If there are restrictions on publicly sharing data—e.g. participant privacy or use of data from a third party—those must be specified.

Reviewer #1: Yes

Reviewer #2: No

5. Is the manuscript presented in an intelligible fashion and written in standard English?

PLOS ONE does not copyedit accepted manuscripts, so the language in submitted articles must be clear, correct, and unambiguous. Any typographical or grammatical errors should be corrected at revision, so please note any specific errors here.

Reviewer #1: Yes

Reviewer #2: No

6. Review Comments to the Author

Please use the space provided to explain your answers to the questions above. You may also include additional comments for the author, including concerns about dual publication, research ethics, or publication ethics. (Please upload your review as an attachment if it exceeds 20,000 characters)

Reviewer #1: (No Response)

Reviewer #2: Thank you for the correction of the manuscript. However, the Discussion part could be even more improved. Not all of the comments have been addressed properly.

Response: Response: Thank you for the comments; we have included study design for studies we have used for comparison purpose. We have omitted “Khat is commonly used in Ethiopia for social and pleasure purposes. Also, certain occupational groups like long-distance truck drivers and students during examination time have a high tendency to consume Khat.” From the discussion section. In the previous comment we have been asked to provide reference; however, since it is our idea we are unable to provide reference as a result we omitted it. (shown in the manuscript with track change.

7
